# Supplementary material for: A Preliminary Study on the Interplay between the Serum Levels of Neurotransmitters and Thyroid Hormones for the Evaluation of the Behavioral Phenotype of Dogs
Source: Animals (Basel). 2023 Jan 26;13(3):411. doi: 10.3390/ani13030411 (PMC9913781; doi:10.3390/ani13030411)
Supplement: Supplementary file 1 [file animals-13-00411-s001.zip › animals-2171540-supplementary.pdf]

The C-BARQ questionnaire

The questions on the following pages are designed to allow you to describe how your dog has been behaving in the recent past. Studies have shown that most of these questions can be grouped or condensed into a set of thirteen major behavioral traits or factors that describe most of the variation in canine temperament, and which are relatively consistent across dogs of different sexes, breeds, and ages:

1. Stranger-directed aggression
2. Owner-directed aggression
3. Dog-directed aggression/fear
4. Trainability
5. Chasing
6. Stranger-directed fear
7. Nonsocial fear
8. Dog-directed fear
9. Separation-related behavior
10. Touch sensitivity
11. Excitability
12. Attachment or attention-seeking
13. Energy

Please try to answer all of the questions. Only use the "not observed/not applicable" option if you have never observed the dog in the situation described.

Section 1: Training and obedience

Some dogs are more trainable than others. By writing in the appropriate number from the scale, please indicate how trainable or obedient your dog has been in each of the following situations in the recent past.

NEVER	SELDOM	SOMETIMES	USUALLY	ALWAYS	Not observed/ Not applicable
0	1	2	3	4	N/A

1. When off the leash, returns immediately when called.	
2. Obeys the "sit" command immediately	
3. Obeys the "stay" command immediately..	
4. Seems to attend/listen closely to everything you say or do .	
5. Slow to respond to correction or punishment; "thick-skinned".	
6. Slow to learn new tricks or tasks.	
7. Easily distracted by interesting sights, sounds, or smells.	
8. Will "fetch" or attempt to fetch sticks, balls, or objects.	

Section 2: Aggression

Some dogs display aggressive behavior from time to time. Typical signs of moderate aggression in dogs include barking, growling and baring teeth. More serious aggression generally includes snapping, lunging, biting, or attempting to bite. By writing in the appropriate number from the scale, please indicate your own dog's recent tendency to display aggressive behavior in each of the following contexts:

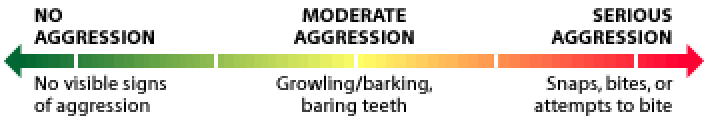

Not observed/ Not applicable

0 1 2 3 4 N/A

9. When verbally corrected or punished (scolded, shouted at, etc.) by you or a household member.	
10. When approached directly by an unfamiliar adult while being walked/exercised on a leash.	
11. When approached directly by an unfamiliar child while being walked/exercised on a leash.	
12. Toward unfamiliar persons approaching the dog while s/he is in your car (at the gas station, for example).	
13. When toys, bones or other objects are taken away by a household member.	
14. When bathed or groomed by a household member.	
15. When an unfamiliar person approaches you or another member of your family at home.	
16. When unfamiliar persons approach you or another member of your family away from your home.	
17. When approached directly by a household member while s/he is eating.	
18. When mailmen or other delivery workers approach your home.	
19. When his/her food is taken away by a household member.	
20. When strangers walk past your home while your dog is outside or in the yard.	
21. When an unfamiliar person tries to touch or pet the dog.	
22. When joggers, cyclists, rollerbladers or skateboarders pass your home while your dog is outside or in the yard.	
23. When approached directly by an unfamiliar male dog while being walked/exercised on a leash.	
24. When approached directly by an unfamiliar female dog while being walked/exercised on a leash.	
25. When stared at directly by a member of the household.	
26. Toward unfamiliar dogs visiting your home.	
27. Toward cats, squirrels or other animals entering your yard.	
28. Toward unfamiliar persons visiting your home.	

29. When barked, growled, or lunged at by another (unfamiliar) dog.	
30. When stepped over by a member of the household.	
31. When you or a household member retrieves food or objects stolen by the dog.	
32. Towards another (familiar) dog in your household.	
33. When approached at a favorite resting/sleeping place by another (familiar) household dog.	
34. When approached while eating by another (familiar) household dog.	
35. When approached while playing with/chewing a favorite toy, bone, object, etc., by another (familiar) household dog.	

Are there any other situations in which your dog is sometimes aggressive? If so, please describe briefly:

Section 3: Fear and anxiety

Dogs sometimes show signs of anxiety or fear when exposed to particular sounds, objects, persons or situations. Typical signs of mild to moderate fear include: avoiding eye contact, avoidance of the feared object, crouching or cringing with tail lowered or tucked between the legs, whimpering and whining, freezing, and shaking and trembling. Extreme fear is characterized by exaggerated cowering, and/or vigorous attempts to escape, retreat or hide from the feared object, person or situation. By writing in the appropriate number from the scale, please indicate your own dog's recent tendency to display fearful behavior in each of the following contexts:

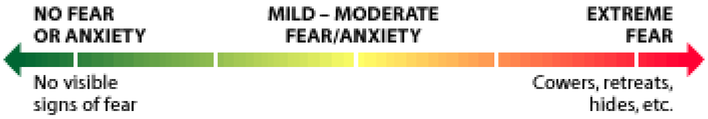

Not observed/
Not applicable

0 1 2 3 4 N/A

36. When approached directly by an unfamiliar adult while away from your home.	
37. When approached directly by an unfamiliar child while away from your home.	
38. In response to sudden or loud noises (e.g. vacuum cleaner, car backfire, road drills, objects being dropped, etc.).	
39. When unfamiliar persons visit your home.	
40. When an unfamiliar person tries to touch or pet the dog.	
41. In heavy traffic.	
42. In response to strange or unfamiliar objects on or near the sidewalk (e.g. plastic trash bags, leaves, litter, flags flapping, etc.).	
43. When examined/treated by a veterinarian.	
44. During thunderstorms, firework displays, or similar events.	

45. When approached directly by an unfamiliar dog of the same or larger size.	
46. When approached directly by an unfamiliar dog of smaller size.	
47. When first exposed to unfamiliar situations (e.g. first car trip, first time in elevator, first visit to veterinarian, etc.).	
48. In response to wind or wind-blown objects.	
49. When having nails clipped by a household member.	
50. When groomed or bathed by a household member.	
51. When stepped over by a member of the household.	
52. When having his/her feet toweled by a member of the household.	
53. When unfamiliar dogs visit your home.	
54. When barked, growled, or lunged at by an unfamiliar dog.	

Section 4: Separation-related behavior

Some dogs show signs of anxiety or abnormal behavior when left alone, even for relatively short periods of time. Thinking back over the recent past, how often has your dog shown each of the following signs of separation-related behavior when left, or about to be left, on its own:

NEVER 0 — SELDOM 1 — SOMETIMES 2 — USUALLY 3 — ALWAYS 4 — Not observed/ Not applicable N/A

55. Shaking, shivering, or trembling.	
56. Excessive salivation.	
57. Restlessness, agitation, or pacing.	
58. Whining.	
59. Barking.	
60. Howling.	
61. Chewing or scratching at doors, floor, windows, curtains, etc.	
62. Loss of appetite.	

Are there any other situations in which your dog is fearful or anxious? If so, please describe briefly:

Section 5: Excitability

Some dogs show relatively little reaction to sudden or potentially exciting events and disturbances in their environment, while others become highly excited at the slightest novelty. Signs of mild to moderate excitability include increased alertness, movement toward the source of novelty, and brief episodes of barking. Extreme excitability is characterized by a general

tendency to over-react. The excitable dog barks or yelps hysterically at the slightest disturbance, rushes toward and around any source of excitement, and is difficult to calm down. By writing in the appropriate number from the scale, please indicate your own dog's recent tendency to become excitable in each of the following contexts:

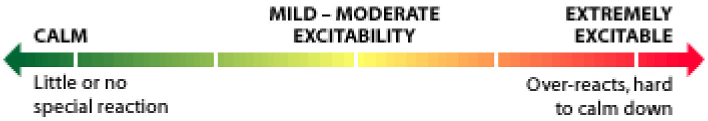

Not observed/ Not applicable

0	1	2	3	4	N/A

63. When you or other members of the household come home after a brief absence.	
64. When playing with you or other members of your household.	
65. When doorbell rings.	
66. Just before being taken for a walk.	
67. Just before being taken on a car trip.	
68. When visitors arrive at your home.	

Are there any other situations in which your dog sometimes becomes over-excited? If so, please describe briefly:

Section 6: Attachment and attention-seeking

Most dogs are strongly attached to their people, and some demand a great deal of attention and affection from them. Thinking back over the recent past, how often has your dog shown each of the following signs of attachment or attention-seeking:

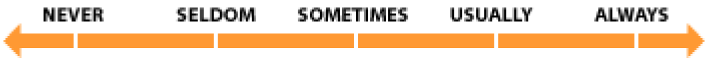

Not observed/ Not applicable

0	1	2	3	4	N/A

69. Displays a strong attachment for one particular member of the household.	
70. Tends to follow you (or other members of the household) about the house, from room to room.	
71. Tends to sit close to, or in contact with, you (or others) when you are sitting down.	
72. Tends to nudge, nuzzle or paw you (or others) for attention when you are sitting down.	
73. Becomes agitated (whines, jumps up, tries to intervene) when you (or others) show affection for another person.	
74. Becomes agitated (whines, jumps up, tries to intervene) when you (or others) show affection for another dog or animal.	

Section 7: Miscellaneous

Dogs display a wide range of miscellaneous behavior problems in addition to those already covered by this questionnaire. Thinking back over the recent past, please indicate how often your dog has shown any of the following behaviors:

NEVER	SELDOM	SOMETIMES	USUALLY	ALWAYS	Not observed/ Not applicable
0	1	2	3	4	N/A

75. Chases or would chase cats given the opportunity.	
76. Chases or would chase birds given the opportunity.	
77. Chases or would chase squirrels, rabbits and other small animals given the opportunity.	
78. Escapes or would escape from home or yard given the chance.	
79. Rolls in animal droppings or other "smelly" substances.	
80. Eats own or other animals' droppings or feces.	
81. Chews inappropriate objects.	
82. "Mounts" objects, furniture, or people.	
83. Begs persistently for food when people are eating.	
84. Steals food.	
85. Nervous or frightened on stairs.	
86. Pulls excessively hard when on the leash.	
87. Urinates against objects/furnishings in your home.	
88. Urinates when approached, petted, handled or picked up.	
89. Urinates when left alone at night, or during the daytime.	
90. Defecates when left alone at night, or during the daytime.	
91. Hyperactive, restless, has trouble settling down.	
92. Playful, puppyish, boisterous.	
93. Active, energetic, always on the go.	
94. Stares intently at nothing visible.	
95. Snaps at (invisible) flies.	
96. Chases own tail/hind end.	
97. Chases/follows shadows, light spots, etc.	
98. Barks persistently when alarmed or excited.	
99. Licks him/herself excessively.	
100. Licks people or objects excessively.	
101. Displays other bizarre, strange, or repetitive behavior(s).*	

*Describe briefly:
